# Nutritional Content of Street Food and Takeaway Food Purchased in Urban Bosnia and Herzegovina

**DOI:** 10.3390/foods10112594

**Published:** 2021-10-27

**Authors:** Sofia Sousa, Inês Lança de Morais, Gabriela Albuquerque, Marcello Gelormini, Mariana Santos, Aida Filipović-Hadžiomeragić, Dragana Stojisavljevic, Albertino Damasceno, Pedro Moreira, João Breda, Nuno Lunet, Patrícia Padrão

**Affiliations:** 1 EPIUnit-Instituto de Saúde Pública, Universidade do Porto, Rua das Taipas 135, 4050-600 Porto, Portugal; sofia.sousa@ispup.up.pt (S.S.); gabriela.albuquerque@ispup.up.pt (G.A.); tino_7117@hotmail.com (A.D.); pedromoreira@fcna.up.pt (P.M.); nlunet@med.up.pt (N.L.); 2 Laboratório para a Investigação Integrativa e Translacional em Saúde Populacional (ITR), Rua das Taipas 135, 4050-600 Porto, Portugal; 3 Faculdade de Ciências da Nutrição e Alimentação da Universidade do Porto, Rua do Campo Alegre 823, 4150-180 Porto, Portugal; 4 Nutrition, Physical Activity and Obesity Programme, Division of Noncommunicable Diseases and Life-Course, World Health Organization (WHO) Regional Office for Europe UN City, Marmorvej 51, 2100 Copenhagen, Denmark; inesbolm@gmail.com (I.L.d.M.); marcello.gelormini@gmail.com (M.G.); 5 Departamento de Alimentação e Nutrição, Instituto Nacional de Saúde Doutor Ricardo Jorge (INSA), Avenida Padre Cruz, 1649-016 Lisboa, Portugal; mariana.coelho@insa.min-saude.pt; 6 Public Health Institute of the Federation of Bosnia and Herzegovina, Tahtali Sokak 17, 71000 Sarajevo, Bosnia and Herzegovina; a.filipovic@zzjzfbih.ba; 7 Public Health Institute of the Republika Srpska, Jovana Dučića 1, 78000 Banja Luka, Bosnia and Herzegovina; dragana.stojisavljevic@med.unibl.org; 8 Faculty of Medicine of the University of Banja Luka, 14 Save Mrkalja, 78000 Banja Luka, Bosnia and Herzegovina; 9 Departamento de Ciências da Saúde Pública e Forenses e Educação Médica, Faculdade de Medicina da Universidade do Porto, Alameda Prof. Hernâni Monteiro, 4200-319 Porto, Portugal; 10 Faculdade de Medicina, Universidade Eduardo Mondlane, Avenida Salvador Allende 702, Maputo 1100, Mozambique; 11 Centro de Investigação em Atividade Física, Saúde e Lazer, Universidade do Porto, Rua Dr. Plácido Costa 91, 4200-450 Porto, Portugal; 12 WHO Regional Office for Europe, 10675 Athens, Greece; rodriguesdasilvabred@who.int

**Keywords:** street food, takeaway food, nutritional value, nutrition transition, Eastern Europe

## Abstract

Street food (SF) and takeaway food (TAF) are important sources of out-of-home meals in urban Bosnia and Herzegovina, where diet-related non-communicable diseases are growing rapidly. This study aimed to characterise SF and TAF purchased in urban areas of Bosnia and Herzegovina, regarding customers’ characteristics and the nutritional composition of the foods and beverages. A cross-sectional study was conducted in Sarajevo and Banja Luka in 2017. SF (*n* = 194) and TAF vending sites (*n* = 154) were selected through random and systematic sampling. Data on the food items purchased and customers’ characteristics were collected by direct observation. Nutritional composition was estimated using data from chemical analyses of the foods most commonly available. Two-thirds of the customers observed (*n* = 755) were aged ≥35 years, half were women and 27.7% were overweight/obese. A total of 929 food items were purchased. The most commonly bought SFs were confectionery (30.5%), water (27.9%) and soft drinks/juices (22.2%). TAF customers purchased mostly savoury pastries (39.8%), breads (27.1%) and main dishes (21.4%). Almost half of customers purchased industrial food (i.e., pre-packaged foods and beverages produced by the food industry). The purchases presented median contents of 18.7 g of fat (39.6% saturated, 32.3% monounsaturated, 22.1% polyunsaturated, 1.5% trans), 838 mg of sodium and 285 mg of potassium. Saturated-fat contribution was higher in SF purchases (60.4% vs. 30.2%, *p* < 0.001), whereas TAF purchases presented higher trans-fat proportion (1.8% vs. 0.6%, *p* < 0.001), sodium (1241 vs. 89 mg, *p* < 0.001) and sodium-potassium ratio (6.1 vs. 0.6, *p* < 0.001). Generally, SF and TAF bought in Sarajevo and Banja Luka were rich in saturated and trans fatty-acids and sodium, and poor in potassium. Nutrition policies promoting use of healthier fats and salt reduction in SF and TAF may contribute to the prevention of diet-related diseases in these settings.

## 1. Introduction

Non-communicable diseases (NCD) are the leading cause of mortality and morbidity worldwide, representing one of the major threats to global sustainable development in the 21st century [1]. In Eastern Europe, NCD are estimated to account for approximately 90% of all deaths in the region, mostly cardiovascular diseases and cancer [2]. The prevalence of obesity in South-Eastern Europe is the highest when compared to the rest of the European sub-regions, with a 30% growth between 2000 and 2014 [3]. In Bosnia and Herzegovina, overweight and obesity affects 60.7% and 26.5% of adults aged 20 years or older, respectively [4] and over one-third are estimated to present high blood pressure [2].

The prevalence of diet-related NCD is rising steeply in low- and middle-income countries (LMIC) [5,6], where Western dietary patterns rich in calorie-dense industrialized foods and beverages are supplanting traditional local diets [7]. In Eastern European countries, the supply of sugar-sweetened beverages and packaged processed foods have increased abruptly [8], as well as the availability of dietary energy, animal fats and sugar [6]. Salt intake is also high in the region, corresponding to approximately twice its maximum dietary recommendation [2].

Nutrition and epidemiological transitions are particularly evident in the most urbanized populations [5,9], where out-of-home eating has become one of the major changes in dietary habits over the last decades [10]. In LMIC, rapid urbanization and economic development have led to a great shift in the lifestyle paradigm of these populations, characterized by physical activity decline, increasing number of hours spent away from home and limited time available for preparation of homemade meals [9]. Eating away from home has been associated with higher energy and fat intake and lower micronutrient ingestion [10], as well as higher risk of becoming overweight or obese [11].

Street food and takeaway food were reported as major sources of ready-to-eat foods and beverages available for consumption away from home, and may play an important role in the foodscape of Bosnia and Herzegovina [12,13]. However, the characterization of nutrition-related research on this type of food sources has been mostly conducted in high-income countries [14,15]. Studies in LMIC are still more centered on hygiene and food safety issues [14,16], and research focusing on consumption of street food and takeaway food in these settings is necessary to support evidence-based nutrition policies. Therefore, this study aims to describe the street food and takeaway food purchases in urban areas of Bosnia and Herzegovina, namely regarding the customers’ characteristics and the nutritional composition of the foods and beverages sold in these venues.

## 2. Methods

This study was implemented in the context of the FEEDCities project, which is based on a stepwise standardized characterization of the food environment in cities from Eastern Europe and Central Asia, as described before [17]. For the purpose of this work, a cross-sectional evaluation of street food and takeaway food customers was carried out between June and August 2017 in Sarajevo and Banja Luka, the two largest urban centres of Bosnia and Herzegovina [18,19].

### 2.1. Eligibility Criteria

In order to select the food vending sites for the study, we adopted the definition of street food proposed by the Food and Agriculture Organization (FAO) and the World Health Organization (WHO), as “ready-to-eat foods and beverages prepared and/or sold by vendors or hawkers especially in the streets and other similar places” [20,21], as well as the definition of a takeaway food vending site proposed by the United Kingdom authorities, as “an outlet whose primary business is the sale of ready-to-eat food and beverages for consumption off the premises” [22]. These included both stationary (street food and takeaway) and mobile (street food) vending units selling food products ready to be consumed immediately without needing further preparation, comprising prepared (e.g., sandwiches), cooked (e.g., main dishes), in natura (e.g., fruits) or industrial foods (e.g., soft drinks, candies). Vending sites selling exclusively non-prepared fruit and vegetables or other not ready-to-eat raw foods (e.g., meat, fish) were not included in the study.

Customers approaching eligible street food or takeaway food vending sites to buy ready-to-eat foods and/or beverages were eligible for the study.

### 2.2. Sampling Procedure

Sampling procedures were designed taking into account the specificities of each city, namely the distribution patterns of street food and takeaway food vending sites observed during preliminary field visits, as previously described in detail [12,13]. In Sarajevo, vending sites were identified within 10 public markets selected in collaboration with local authorities. A study area was defined within each market, corresponding to 500 m diameter buffers around the centre of the selected markets, with the exception of the larger main city market (*Markale*), where a 1500 m diameter was defined, to accommodate its wider distribution of vending sites. In Banja Luka, the vending sites were mainly clustered in the main city market (*Gradska tržnica*), as well as around public transportation stops (bus stations). Thus, the main city market was selected along with 10 bus stops, each of which corresponded to the main station of the city bus routes. To define the study area, a 1500 m buffer was drawn around the centroid of the main city market; and for each bus stop, the buffer diameter was 100 m.

Field researchers working in pairs canvassed all publicly accessible streets within the study areas of each city in order to identify eligible vending sites. All vending sites identified in each city were selected for this study.

In each vending site, all customers buying any food or beverage meeting the abovementioned criteria, were observed; the period of observation started at the next multiple of five minutes and ended after 10 min or when four customers were observed, whichever came first. If no customer was observed during this period, field researchers would move on to the next vending site. Observations were performed both on week days and weekends and covering all businesses’ working hours (from 8 a.m. to 6 p.m.).

### 2.3. Data Collection

Data on customers’ characteristics and their street food or takeaway food purchases were collected through direct observation, at each identified vending site and performed independently by two local researchers, placed at a distance considered sufficient not to compromise the normal behaviour of the customers nor the regular activity of the vending sites. To standardize and improve the accuracy of the anthropometric evaluation by direct observation, the observers were trained using the Body Mass Index (BMI)-based body size guides for women and men by Harris et al. [23].

The two observers registered the type of vending site (street food or takeaway) and described, for each customer approaching the venue, the foods and/or beverages purchased (hereafter referred to as food items) and their quantities, as well as customers’ characteristics, including sex and estimated age (in years: <18, (18–25), (25–35), (35–45), (45–65) or ≥65) and BMI-based weight status (underweight, normal weight, overweight or obese). The inter-observer concordance was high to very high regarding both the customers’ characteristics and the food items purchased (Supplementary Table S1). Data on age and weight status was considered for data analysis only for the customers regarding whom there was agreement between observers (*n* = 701 and *n* = 683, respectively). For the food items and quantities purchased, a set of decision rules was implemented in order to eliminate conflicts of observation and uniform the purchases (Supplementary Table S2).

The food items purchased were classified into homemade (foods and beverages prepared and/or cooked at home or in the venue, even if using industrial ingredients) or industrial (foods and beverages produced by the food industry and sold as such, with no further preparation). Based on the WHO nutrient profile model [24], the food items were also grouped into seven sub-categories of foods (1) Main dishes; (2) Breads; (3) Savoury pastries; (4) Savoury snacks; (5) Buns, cakes and cookies; (6) Ice-cream, chocolate and confectionery; and (7) Sandwiches; and five sub-categories of beverages (1) Coffee; (2) Water; (3) Soft drinks and industrial juices; (4) Alcoholic beverages; and (5) Yoghurt.

### 2.4. Nutritional Composition Estimation

The foods and beverages most frequently available at street food and takeaway food vending sites from each city were identified in a preceding step of the FEEDCities project [12,13]. Then, in each setting, the 20 most frequently available homemade foods and the 10 most frequently available industrial foods were selected for laboratorial analysis. Common foods and beverages with known nutritional composition, such as coffee, soft drinks, water and fruit, were excluded from this selection.

In each city, four samples of each food were collected as part of a regular transaction in different randomly selected vending sites. Chemical analyses, including total fat, fatty acids, sodium and potassium, were performed in accordance with standardized and recommended procedures [25], and described elsewhere [12,13].

For the description of the nutritional composition of the purchases, it was considered a sub-sample (*n* = 474) of customers who purchased exclusively foods which were chemically analysed; customers who, in addition to those foods, bought one or more beverages of known composition (water, coffee and soft drinks) were also included in this sub-sample. A street food or takeaway food purchase was defined as an acquisition consisting of at least one food. As such, customers buying only beverages, as well as those who purchased at least one food item without nutritional composition data, were excluded. No statistically significant differences were found between the included and excluded customers, regarding sex, age or weight status.

The nutritional value of the street food and takeaway food purchases of each customer was then computed by adding up the estimated contents of total fat, fatty acids, sodium and potassium of all food items purchased by the same customer in a single occasion. For each purchase, percentages of the WHO daily recommendations were computed assuming an average adult with a daily reference intake of 2000 kcal of total energy value (TEV): saturated fatty acids (SFA), <10% TEV [26]; trans fatty acids (TFA), <1% TEV [27]; sodium, <2000 mg [28]; potassium, ≥3510 mg [29]; sodium to potassium molar ratio, 1 [28,29]. For all fatty acids, contents were also computed as proportions of the total amount of fat, and were expressed as g/100 g of total fat.

### 2.5. Data Analysis

Absolute and relative frequencies were used to describe the customers and their purchases, and the nutritional value of the purchases was described using medians and percentiles 25 and 75. For the description of the sample and the comparison of purchases between groups, the variables corresponding to the customers’ characteristics were dichotomized (sex: male or female; age: <35 years or ≥35 years; weight status: underweight/normal weight or overweight/obesity). Inter-observer concordance for the demographic and anthropometric characteristics of the customers, as well as the food items purchased and its quantities, was assessed through percentage of agreement and Cohen’s kappa coefficient with 95% confidence interval. Pearson’s Chi-squared and Mann-Whitney’s U tests were used to compare the frequencies of the food items purchased and the nutritional content of the purchases, respectively, regarding demographic and anthropometric characteristics of the customers observed and type of vending site frequented. Differences were considered statistically significant when the critical level of significance (*p*) was less than 0.05. Statistical analyses were performed using Stata^®^ version 15.0 (StataCorp., College Station, TX, USA).

## 3. Results

A total of 348 vending sites were observed (*n* = 194 street food; *n* = 154 takeaway), of which 267 (76.7%) had customers observed. A total of 755 customers were observed: 499 (66.1%) in Sarajevo and 457 (60.5%) takeaway food customers. Demographic and anthropometric characteristics of the individuals observed, as well as some main aspects regarding the food items purchased, are presented in Table 1. Approximately half of the customers (50.6%) were female, two-thirds (66.8%) were aged 35 years and older and almost one-third (27.7%) were overweight or obese. Most customers (79.1%) purchased only one food item and more than two-thirds (68.1%) purchased only foods. Almost half (43.7%) of the customers purchased at least one industrial food item; this frequency was significantly higher for street food customers (78.2% vs. 21.2%, *p* < 0.001).

**Figure 1:**
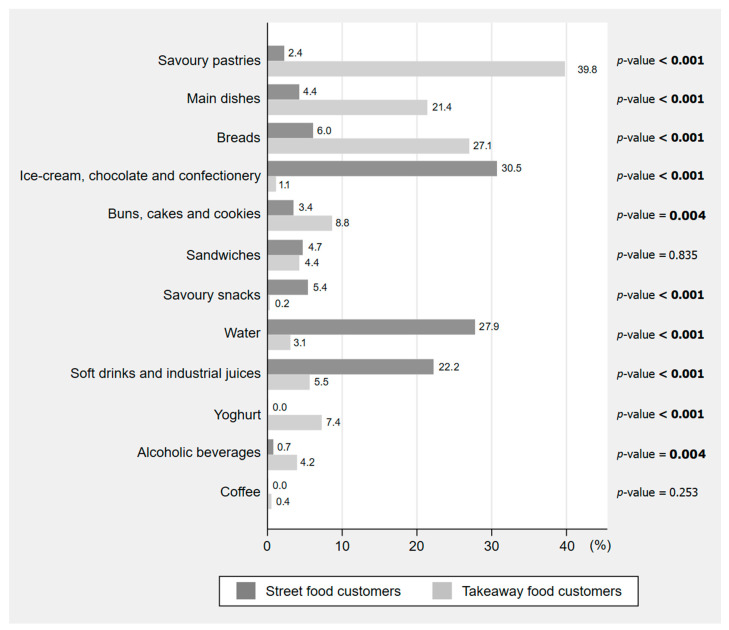
Ready-to-eat foods and beverages purchased by the customers observed in street food and takeaway food vending sites in Sarajevo and Banja Luka, Bosnia and Herzegovina (*n* = 755). The proportions presented are relative to the total number of customers from each type of vending site (street food: *n* = 298; takeaway food: *n* = 457). The sum of the percentages may exceed 100% because the same customer could buy more than one food or beverage. Values in bold represent statistically significant differences according to Pearson’s Chi-squared test with a significance level of 0.05.

A total of 929 food items were purchased, corresponding to an average of 1.2 food items per customer and 5.8 food items per 10 min of observation. The most commonly purchased takeaway foods were savoury pastries (39.8% of all takeaway customers), breads (27.1%) and main dishes (21.4%), while street food customers bought mostly ice-cream, chocolate and confectionery (30.5% of all street food customers), water (27.9%) and soft drinks and industrial juices (22.2%) (Figure 1). The frequencies of foods and beverages purchased by sex, age and weight status of the customers observed are presented in Supplementary Table S3.

The median total fat content of one purchase was 18.7 g, with SFA and TFA median contents accounting for 38.0% and 8.1% of their recommendations, respectively. The median sodium content was 838 mg, which accounted for 41.9% of its maximum daily intake recommendation, while supplying 285 mg of potassium (8.1% of its minimum recommendation). In comparison to street food, takeaway food purchases presented higher median contents of total fat (22.0 vs. 13.9 g, *p* < 0.001) as well as all fatty acids. SFA and TFA median contents in takeaway food purchases accounted for 39.7% and 21.4% of their maximum daily intake recommendations, respectively. Takeaway food customers also presented purchases with higher median contents of sodium (1241 vs. 89 mg, *p* < 0.001) and potassium (323 vs. 162 mg, *p* < 0.001). Regarding nutrient density (per 100 g), street food purchases presented higher total fat (12.6 vs. 8.8 g/100 g, *p* < 0.001), SFA (8.9 vs. 3.0 g/100 g, *p* < 0.001) and potassium (216 vs. 161 mg/100 g, *p* < 0.001), while takeaway food customers presented purchases with higher polyunsaturated fatty acids (PUFA) (1.9 vs. 1.0 g/100 g, *p* < 0.001), TFA (0.19 vs. 0.11 g/100 g, *p* < 0.001) and sodium levels (565 vs. 117 mg/100 g, *p* < 0.001). Sodium to potassium ratio was significantly higher in takeaway food purchases (6.1 vs. 0.6, *p* < 0.001) (Table 2).

**Figure 2:**
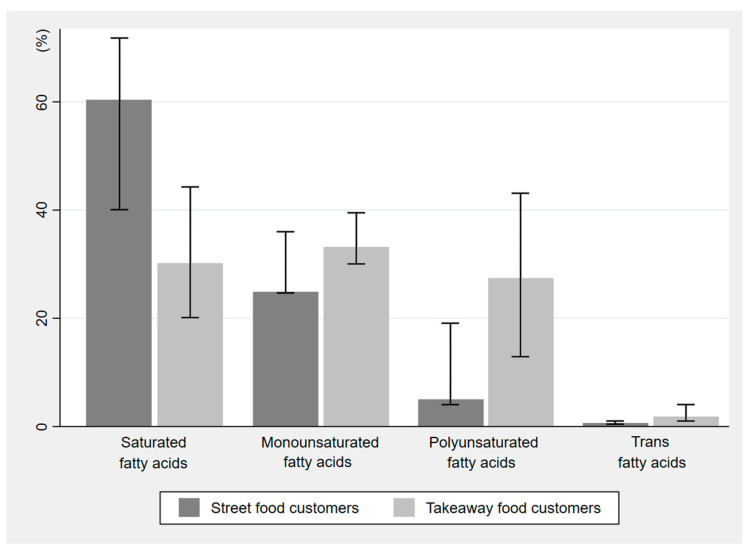
Estimated fatty-acid contents (g/100 g of total fat) of the purchases made by a sub-sample (*n* = 474) of street food and takeaway food customers observed in Sarajevo and Banja Luka, Bosnia and Herzegovina. The values shown are presented as median and inter-quartile range.

Regarding lipid profile, the highest median contribution to total fat came from SFA (39.6 g/100 g total fat), followed by monounsaturated fatty acids (MUFA) (32.3 g/100 g total fat), PUFA (22.1 g/100 g total fat) and TFA (1.5 g/100 g total fat). Purchases from street food customers presented higher contribution from SFA (60.4 vs. 30.2 g/100 g total fat, *p* < 0.001) and lower proportions of PUFA (5.0 vs. 27.4 g/100 g total fat, *p* < 0.001), whereas takeaway food customers presented purchases with higher TFA levels (1.8 vs. 0.6 g/100 g total fat, *p* < 0.001) (Figure 2). Purchases containing at least one industrial food item had a higher proportion of SFA (58.9 vs. 30.0 g/100 g of total fat, *p* < 0.001) and lower contribution from PUFA (5.0 vs. 28.2 g/100 g of total fat, *p* < 0.001) and MUFA (30.2 vs. 32.5 g/100 g of total fat, *p* = 0.034) than those with no industrial food items.

The nutritional composition of the purchases by sex, age and weight status of the customers is presented in Table 3. Male customers presented purchases with higher serving size, total fat, SFA, TFA, MUFA, sodium and potassium. Older customers presented purchases richer in PUFA, n-6 fatty acids and sodium. Purchases from overweight/obese customers showed higher level of TFA and sodium. Considering nutritional composition per 100 g, men and older customers presented purchases richer in sodium, while younger individuals presented purchases with higher levels of total fat, SFA and MUFA.

## 4. Discussion

In the two main urban areas of Bosnia and Herzegovina, the purchase of street food and takeaway food was frequent, as demonstrated by the large number of foods and beverages bought within a limited observation period. The food items identified varied according to the type of vending site: takeaway food purchases consisted mainly of pastries, bread and main dishes, whereas those from street food customers included mostly industrial sweets and beverages. As a result, the nutritional composition of the purchases in each type of venue also differed. Generally, in takeaway food vending sites purchases presented higher total fat content, trans-fat proportion and sodium to potassium ratio, while street food purchases showed higher contribution from saturated fats.

The acquisition of homemade food items was commonly observed, with traditional foods such as *cevapi* (seasoned minced meat rolled into small sausages and served with onions in a traditional bread), *burek* and *sirnica* (traditional savoury baked pies, usually filled with beef and cheese, respectively) and *kifla* (local bread) being amongst the most frequently bought. This suggests that street-vended local foods continue to have cultural expression and dietary importance to these populations, as observed in other contexts [16,30]. However, it should be noted that almost half of the customers bought industrial food items, mostly ice-cream, chocolate and confectionery, chips and soft drinks. Purchases containing industrial food items presented a worse lipid profile than those with no industrial food items, which is in line with previous evidence showing that this type of food products is usually rich in fat, mostly saturated [31]. It has been observed that ultra-processed foods compromise the quality of the diet [32,33] and greatly contribute to excess weight gain and its comorbidities [34,35], and there is a margin for the food industry to improve the nutritional quality of its products, by using healthier recipes and ingredients. Also, promoting higher availability and affordability of healthier street-vended food options, such as ready-to-eat fruits, vegetables, legumes and whole grains, could contribute to counteract the widespread access to less healthy foods, ultimately influencing positively the consumers’ choices, as seen in other contexts [36,37].

The street food and takeaway food purchases presented considerable quantities of total fat, SFA and TFA per serving. These values were particularly high in purchases from takeaway venues, reaching almost 40% of the WHO recommendation for SFA [26], and exceeding 20% of the WHO recommendation for TFA [27]. However, it is noteworthy that the amounts purchased by takeaway food customers were much larger when compared to those bought by street food customers. When analysing the nutritional value per 100 g of purchase, it was possible to observe that both total and saturated fat contents were significantly higher in street food, when compared to takeaway purchases. Considering the lipid profile per 100 g of total fat, it was observed that in both types of venue the highest contribution came from SFA rather than unsaturated fats; TFA also accounted for an important share of the purchases’ total fat. In each type of vending site, it was possible to identify some concerning results: street food purchases presented very high proportions of SFA (exceeding 60% of total fat) as opposed to the very small contributions of PUFA, whereas takeaway customers presented purchases with high contents in TFA, with its median almost reaching the limit of 2 g/100 g of total fat recommended by the WHO Europe [38] and most recently legislated for the European Union [39]. From all food items analysed for TFA, approximately one in every three (33.8%) exceeded this limit, showing that efforts must be undertaken in order to lower contents of industrially-produced TFA through reformulation of industrial foods. This, coupled with continuous monitoring, would be essential in the achievement of this goal. It was also observed that, from a single purchase, more than half of the customers (53.4%) exceeded 50% of the maximum recommendation for SFA and almost half (46.4%) exceeded 50% of the maximum recommendation for TFA, suggesting that the customers observed are likely to surpass the daily recommended amounts for these fatty acids when consuming one or more meals from a street food or takeaway food vending site. In other settings, high contents of total, saturated and trans-fat have also been documented in both street [16,30,40] and takeaway food [41,42], and their frequent consumption have been associated with negative health outcomes [43,44]. In this study, homemade foods were identified as major sources of TFA, which might suggest a common use of cooking fats and shortenings containing trans-fat, or unhealthy cooking methods, such as frying, in the preparation and confection of these foods [12,13]. This underlines the need for interventions aimed at improving the ingredients and culinary techniques used, for example through price policies that promote the purchase of healthier ingredients, or through nutrition education programmes aimed at increasing vendors’ knowledge and skills related to healthier cooking practices. A systematic review by Downs et al., assessing the impact of policies to reduce trans-fat consumption, reported a reduction on cardiovascular mortality ranging from 1.3% to 6.4% attributable to TFA policies (including voluntary limits on TFA, TFA labelling and legislative bans) [45]. Although no study was conducted in Bosnia and Herzegovina, these results indicate that strategies aimed at TFA reduction from the food supply are expected to decrease the burden of diet-related disease.

Our findings also showed that one single purchase provided almost half of the maximum daily intake recommendation for sodium [28], with higher values in the case of takeaway food. The discrepancies in the nature of the foods available in each type of vending site justify the differences found, since the street food bought consisted mainly of industrial sweets and drinks, which are not relevant sources of sodium. It was also observed that nearly half the customers exceeded its limit of 2000 mg in a single purchase. On the other hand, both street food and takeaway food purchases appeared to be very poor sources of potassium, which can be attributed in part to the low content in fruits, vegetables and pulses of the foods purchased. Sodium to potassium ratio exceeded five times the WHO recommendation [28,29], and this was observed even in purchases containing foods which could be considered as main courses, where a higher amount of potassium would be expected. Homemade food items presented a median sodium to potassium ratio significantly higher than the industrial ones (6.8 vs. 0.5, *p* < 0.001), which can be explained by the excessive addition of salt and/or sodium-rich ingredients, as reported in other settings [46,47]. Results from the Global Burden of Disease study 2019 showed that high blood pressure is the leading risk factor for disease in Eastern Europe, also underscoring the disproportionately high burden of cardiovascular disease in this region [48]. Both reducing dietary sodium content and increasing potassium intake have shown documented benefits to cardiovascular health [49,50]. A study modelling the impact of salt reduction strategies in 23 LMIC showed that a reduction in salt intake to 5 g/day over 10 years could correspond to a potential impact of 23.7% of deaths averted due to cardiovascular disease [51,52]. Salt reduction policies have also been identified as one of the most cost-effective measures to improve populations’ health [51]. However, to the best of our knowledge, no salt reduction initiatives have been implemented in this country [4], thus highlighting an opportunity for action. Public health strategies may include the development of interventions encouraging sellers and small manufacturers to use healthier ingredients and to adopt cooking practices for reducing the addition of salt into homemade foods, as well as the implementation of legislation limiting the amount of salt in the industrial ingredients used during preparation of these foods. Increasing the availability of fruits and vegetables as snacks ready for consumption, as well as encouraging the inclusion of more vegetables in the culinary preparations to be sold in the streets, could also help improve sodium to potassium balance of the street food and takeaway meals consumed in these settings.

Although we were unable to analyse sugar contents of the samples collected, it was possible to observe that sugary foods and beverages (i.e., sweet pastries, confectionery, soft drinks and industrial juices) were purchased by a relevant part of our sample (232 customers, 30.7% of total). Accordingly, a high availability of cookies, cakes, industrial sweets, soft drinks and fruit-juice based beverages has been previously reported in these cities [12,13]. The consumption of sugar-rich foods and beverages has been linked to a higher risk of obesity and other cardio-metabolic diseases [53,54]. WHO guidelines recommend adults and children to reduce their daily intake of free sugars to less than 10% of their total energy daily intake; and further reduction to below 5% would provide additional health benefits [55].

Regarding customers’ characteristics, male customers and those classified as overweight/obese presented purchases with generally higher levels of fat (total, saturated and trans) and sodium than women and normal-weighted customers. Energy requirements of men are generally higher than those of women, which may partly justify the higher amounts of food purchased, and consequently the higher contents of fats and sodium. However, high intakes of SFA, TFA and sodium have documented deleterious effects on health [27,28,50], being particularly concerning among individuals that already suffer from overweight or obesity. Strategies aimed at improving consumption patterns with a focus on the prevention of diet-related diseases would be of particular importance in these groups.

Some limitations and strengths of the present study should be discussed. Estimation of weight status was performed through direct observation, which still lacks validation in these populations. However, agreement between observers was high, regarding not only sex, age and weight status of the customers, but also the foods and beverages bought and their quantities, resulting in consistent and reliable observational data. This was achieved due to successful training, which included a clear definition of the research goals and standardized procedures, followed by practical application with continuous supervision. Although body measurement is currently used as the gold standard, it was considered that this method could pose some practical difficulties to data collection. The need for additional human and material resources, namely numerous calibrated body scales, stadiometers, as well as measurement rooms for individual privacy, could create a large apparatus around the vendors, disturbing their regular activity. Also, frequent refusal to participate would be expected due to individual and/or cultural barriers to body measurement. Future work should consider the validation of the estimation of weight status through direct observation by trained researchers, as this would constitute a more practical and culture-friendly method to collect accurate anthropometric data. Another factor which could present potential additional challenges to anthropometric evaluation was the weather, since climate variations throughout the study could lead to changes in the customers’ clothing. In this study, both cities were assessed during summer, that way minimising errors associated to clothing. Also, in both cities, the specific tasks related to data collection were carried out in the month of July, during which weather conditions were favourable, with no rain and small temperature variations [56,57]. Another limitation of this study is the fact that we cannot ensure that all customers bought food items only for themselves, and for one single meal. However, the number of food items purchased per customer, which was close to one, suggested that this assumption should be correct in the majority of cases. Furthermore, the assessment of the purchases as a proxy measure to consumption, using direct observation rather than interview, allowed us to avoid potential behavioural or social desirability biases. Nutritional composition characterization included the lipid profile, sodium and potassium contents through laboratorial analysis of the most common foods available. This constitutes innovative and accurate information on these key nutrients for diet-related NCD, hence contributing to a better understanding of the health implications of street food and takeaway food frequent consumption in these cities. Finally, the methodology used in this study allows the standardization of the evaluations and, thus, valid comparisons among different cities or countries, although generalizability is limited due to local cultural specificities.

In conclusion, street and takeaway foods are commonly bought in these urban areas of Bosnia and Herzegovina. Purchases are characterized by the coexistence of globalized industrial food products with local and traditional foods and beverages. Customers presented purchases generally rich in SFA, TFA and sodium, and poor in potassium, which may impact negatively on health. Nutritional policies targeting the improvement of the nutritional profile identified in the street food and takeaway food purchased, namely towards the reduction of TFA, sodium and/or sugar contents, as well as the modulation of the availability of highly energy-dense foods, have the potential to reduce mortality from ischemic heart disease and stroke, which are the two leading causes of death in Bosnia and Herzegovina, and to reduce the burden of risk factors such as hyperglycaemia, high blood pressure, high BMI, dietary factors and dyslipidemia, which constitute five of the top seven risk factors for NCD mortality and disability in the country [48]. Political efforts should follow a multi-front approach, targeting simultaneously the urban food environment, the food industry and the population. Environmental strategies in combination with policy and educational programmes have been documented as effective in preventing obesity and other diet-related chronic diseases in low-income settings [58]. Public health strategies should concern not only the consumers as the recipients of the foods, but also the vendors and manufacturers as the vectors of the nutritional quality of the foods publicly available for consumption, and could be incorporated into existing programmes towards nutrition-related NCD prevention.

## Figures and Tables

**Table 1:** Customer’s demographic and anthropometric characteristics, and items purchased, estimated by direct observation of street food and takeaway food vending sites in Sarajevo and Banja Luka, Bosnia and Herzegovina.

	Total	Street Food Customers	Takeaway Food Customers	*p*
	*n* = 755	*n* = 298	*n* = 457	
	*n* (%)	*n* (%)	*n* (%)	
Sex				**0.010**
Male	373 (49.4)	130 (43.6)	243 (53.2)	
Female	382 (50.6)	168 (56.4)	214 (46.8)	
Age ^a^				**<0.001**
<35 years	233 (33.2)	120 (44.1)	113 (26.3)	
≥35 years	468 (66.8)	152 (55.9)	316 (73.7)	
Weight status ^a^				0.105
Underweight/Normal weight	494 (72.3)	206 (75.7)	288 (70.1)	
Overweight/obesity	189 (27.7)	66 (24.3)	123 (29.9)	
Number of items purchased				
1 (median)	597 (79.1)	272 (91.3)	325 (71.1)	**<0.001**
>1	158 (20.9)	26 (8.7)	132 (28.9)	
Purchased foods or beverages?				**<0.001**
Only foods	514 (68.1)	150 (50.3)	364 (79.7)	
Only beverages	153 (20.3)	130 (43.6)	23 (5.0)	
Foods and beverages	88 (11.6)	18 (6.0)	70 (15.3)	
Purchased homemade or industrial?				**<0.001**
Only homemade	425 (56.3)	65 (21.8)	360 (78.8)	
Only industrial	257 (34.0)	231 (77.5)	26 (5.7)	
Homemade and industrial	73 (9.7)	2 (0.7)	71 (15.5)	

^a^ For the variables age and weight status, the data presented corresponds to the customers in which there was agreement between observers (*n* = 701 and *n* = 683, respectively). Values in bold represent statistically significant differences according to Pearson’s Chi-squared test with a significance level of 0.05.

**Table 2:** Estimated fatty acids, sodium and potassium contents of the purchases made by a sub-sample (*n* = 474 ^a^) of street food and takeaway food customers observed in Sarajevo and Banja Luka, Bosnia and Herzegovina.

	Total	Street Food Customers	Takeaway Food Customers	*p*
	*n* = 474	*n* = 127	*n* = 347	
	Median (P25–P75)	Median (P25–P75)	Median (P25–P75)	
Amount purchased (g)	159 (83–294)	66 (66–192)	179 (96–318)	**<0.001**
Per total purchase				
Total fat (g)	18.7 (7.7–32.0)	13.9 (8.2–18.9)	22.0 (6.6–37.2)	**<0.001**
SFA (g)	8.4 (1.9–12.5)	7.4 (4.0–9.2)	8.8 (1.2–14.4)	0.546
MUFA (g)	5.7 (1.6–10.3)	3.3 (1.6–6.6)	7.7 (1.8–12.5)	**<0.001**
PUFA (g)	2.6 (1.4–6.0)	0.8 (0.6–1.6)	4.0 (1.7–6.7)	**<0.001**
n-6 (g)	2.5 (1.3–5.8)	0.8 (0.6–1.5)	3.8 (1.4–6.6)	**<0.001**
n-3 (g)	0.1 (0.1–0.2)	0.1 (0.0–0.1)	0.1 (0.1–0.3)	**<0.001**
TFA (g)	0.18 (0.05–0.92)	0.08 (0.04–0.17)	0.47 (0.05–1.16)	**<0.001**
Na (mg)	838 (365–1934)	89 (39–490)	1241 (540–2140)	**<0.001**
K (mg)	285 (154–500)	162 (143–292)	323 (154–536)	**<0.001**
Per 100 g of purchase				
Total fat (g)	9.5 (6.0–17.6)	12.6 (7.1–21.1)	8.8 (5.5–15.0)	**<0.001**
SFA (g)	3.3 (1.6–7.0)	8.9 (2.7–14.0)	3.0 (1.2–5.3)	**<0.001**
MUFA (g)	3.0 (1.9–5.0)	3.0 (2.1–5.0)	3.0 (1.8–4.8)	0.352
PUFA (g)	1.8 (1.0–3.0)	1.0 (0.4–2.0)	1.9 (1.1–3.6)	**<0.001**
n-6 (g)	1.5 (0.9–2.9)	0.9 (0.4–1.7)	1.8 (1.0–3.3)	**<0.001**
n-3 (g)	0.1 (0.0–0.1)	0.1 (0.0–0.1)	0.1 (0.0–0.2)	**0.030**
TFA (g)	0.16 (0.06–0.28)	0.11 (0.03–0.19)	0.19 (0.07–0.32)	**<0.001**
Na (mg)	562 (357–674)	117 (59–523)	565 (451–758)	**<0.001**
K (mg)	169 (118–232)	216 (144–273)	161 (116–201)	**<0.001**
Na/K ratio	5.9 (4.8–7.2)	0.6 (0.5–5.8)	6.1 (5.6–7.4)	**<0.001**

SFA, saturated fatty acids; MUFA, monounsaturated fatty acids; PUFA, polyunsaturated fatty acids; TFA, trans fatty acids; Na, sodium; K, potassium. Values in bold represent statistically significant differences according to Mann-Whitney’s U test with a significance level of 0.05. ^a^ Only customers purchasing foods with laboratorial data and beverages (when applicable) of known composition (i.e., water, coffee and soft drinks) were included. Customers purchasing only beverages were not included.

**Table 3:** Estimated fatty acids, sodium and potassium contents of the purchases made by a sub-sample (*n* = 474 ^a^) of street food and takeaway food customers observed in Sarajevo and Banja Luka, Bosnia and Herzegovina, by sex, age and weight status.

	Sex			Age ^b^			Weight Status ^b^		
	Male *n* = 229	Female *n* = 245	*p*	<35 Years *n* = 141	≥35 Years *n* = 308	*p*	Underweight/ Normal Weight *n* = 311	Overweight/ Obesity *n* = 120	*p*
	Median (P25–P75)			Median (P25–P75)			Median (P25–P75)		
Amount purchased (g)	179 (89–318)	108 (66–247)	0.004	154 (66–247)	171 (83–318)	0.155	145 (82–247)	191 (83–318)	0.126
Per total purchase									
Total fat (g)	22.0 (8.8–35.7)	16.0 (6.9–27.9)	**0.017**	17.8 (9.1–27.8)	19.1 (6.6–32.3)	0.967	17.8 (8.0–30.6)	21.7 (7.7–34.0)	0.269
SFA (g)	9.1 (4.0–14.4)	7.3 (1.3–11.8)	**0.020**	9.1 (4.2–12.5)	8.1 (1.11–12.5)	0.254	8.4 (3.1–12.0)	8.8 (1.8–16.8)	0.263
MUFA (g)	7.1 (3.0–11.5)	4.8 (1.5–9.4)	**0.011**	5.6 (3.3–9.5)	6.6 (1.4–11.3)	0.912	5.6 (1.6–9.8)	6.6 (1.8–11.8)	0.341
PUFA (g)	2.9 (1.4–6.1)	2.0 (1.3–5.4)	0.192	1.9 (1.0–4.5)	3.0 (1.4–6.4)	**0.022**	2.4 (1.4–6.0)	2.7 (1.4–5.3)	0.745
n-6 (g)	2.8 (1.3–6.0)	1.9 (1.1–5.1)	0.211	1.9 (0.9–4.3)	2.8 (1.3–6.3)	**0.023**	2.2 (1.3–5.8)	2.5 (1.3–5.2)	0.649
n-3 (g)	0.1 (0.1–0.2)	0.1 (0.1–0.2)	0.726	0.1 (0.1–0.2)	0.1 (0.1–0.2)	0.145	0.1 (0.1–0.2)	0.1 (0.1–0.2)	0.751
TFA (g)	0.34 (0.05–0.93)	0.16 (0.04–0.88)	**0.013**	0.18 (0.05–0.78)	0.25 (0.04–0.92)	0.987	0.18 (0.04–0.92)	0.31 (0.05–1.06)	**0.021**
Na (mg)	1122 (459–2140)	540 (260–1561)	**<0.001**	548 (237–1416)	1080 (459–2085)	**0.010**	712 (308–1751)	1122 (412–2140)	**0.018**
K (mg)	308 (155–511)	235 (143–430)	**<0.001**	256 (150–437)	289 (154–507)	0.095	256 (154–471)	324 (158–509)	0.079
Per 100 g of purchase									
Total fat (g)	9.0 (6.0–17.6)	10.1 (5.5–17.0)	0.963	10.3 (7.2–20.3)	9.0 (4.9–15.1)	**0.018**	9.4 (6.0–18.1)	9.5 (5.5–15.3)	0.552
SFA (g)	3.3 (1.7–7.1)	3.3 (1.3–6.8)	0.589	4.5 (2.6–9.2)	3.1 (1.1–6.0)	**0.001**	3.3 (1.6–8.2)	3.3 (1.3–6.0)	0.561
MUFA (g)	3.0 (2.0–5.0)	2.9 (1.8–5.0)	0.529	3.3 (2.2–5.3)	2.7 (1.8–5.0)	**0.033**	2.9 (2.0–5.0)	3.0 (1.8–4.8)	0.663
PUFA (g)	1.8 (0.9–3.0)	1.8 (1.0–3.2)	0.389	1.5 (1.0–2.9)	1.8 (1.0–3.0)	0.155	1.8 (1.0–3.0)	1.7 (0.8–2.8)	0.489
n-6 (g)	1.5 (0.9–2.8)	1.5 (0.9–3.2)	0.433	1.5 (0.9–2.7)	1.6 (0.9–3.0)	0.190	1.5 (0.9–3.0)	1.5 (0.8–2.7)	0.529
n-3 (g)	0.1 (0.0–0.1)	0.1 (0.0–0.2)	0.080	0.1 (0.0–0.1)	0.1 (0.0–0.1)	0.863	0.1 (0.0–0.1)	0.0 (0.0–0.1)	0.124
TFA (g)	0.16 (0.06–0.31)	0.14 (0.05–0.26)	0.152	0.18 (0.07–0.26)	0.16 (0.05–0.30)	0.410	0.14 (0.06–0.26)	0.18 (0.05–0.31)	0.378
Na (mg)	565 (393–689)	517 (317–629)	**0.024**	522 (245–629)	562 (397–689)	**0.049**	541 (331–627)	562 (376–758)	0.181
K (mg)	169 (120–232)	169 (116–232)	0.709	172 (120–243)	169 (116–232)	0.429	167 (118–225)	172 (116–242)	0.657
Na/K ratio	6.0 (4.9–7.2)	5.8 (3.7–7.0)	0.063	5.8 (1.6–7.2)	5.9 (4.9–7.2)	0.065	5.9 (4.3–7.2)	5.9 (4.8–7.2)	0.425

SFA, saturated fatty acids; PUFA, polyunsaturated fatty acids; TFA, trans fatty acids; MUFA, monounsaturated fatty acids; K, potassium; Na, sodium. Values in bold represent statistically significant differences according to Mann Whitney’s U test with a significance level of 0.05. ^a^ Only customers purchasing foods with laboratorial data and beverages (when applicable) of known composition (i.e., water, coffee and soft drinks) were included. Customers purchasing only beverages were not included; ^b^ For the variables age and weight status, the data presented corresponds to the customers in which there was agreement between observers (*n* = 449 and *n* = 431, respectively).

## Data Availability

The data presented in this study are available upon request to the corresponding author.

## Supplementary Materials

The following are available online at https://www.mdpi.com/article/10.3390/foods10112594/s1, Table S1: Inter-observer concordance on demographic and anthropometric characteristics and items purchased by the customers observed in street food and takeaway food vending sites in Bosnia and Herzegovina. Table S2: Decision rules for the elimination of conflicts of observation. Table S3: Ready-to-eat foods and beverages purchased by the customers observed in street food and takeaway food vending sites in Bosnia and Herzegovina, overall and by sex, age and weight status.
