# Supplementary material for: Nutritional Content of Street Food and Takeaway Food Purchased in Urban Bosnia and Herzegovina
Source: Foods. 2021 Oct 27;10(11):2594. doi: 10.3390/foods10112594 (PMC8620844; doi:10.3390/foods10112594)
Supplement: Supplementary file 1 [file foods-10-02594-s001.zip › foods-1433842-supplementary.pdf]

Supplementary Material

Supplementary Table S1. Inter-observer concordance on demographic and anthropometric characteristics and items purchased by the customers observed in street food and takeaway food vending sites in Bosnia and Herzegovina.

	**Total**		**Street food**		**Takeaway food**	
	**% agreement**	**Cohen's Kappa (95% CI)**	**% agreement**	**Cohen's Kappa (95% CI)**	**% agreement**	**Cohen's Kappa (95% CI)**
**CUSTOMERS**	***n* = 755**		***n* = 298**		***n* = 4 57**	
**Sex**						
Male<br>Female	100.0%	1.00 (0.93-1.00)	100.0%	1.00 (0.89-1.00)	100.0%	1.00 (0.91-1.00)
**Age**						
<35 years<br>≥35 years	92.9%	0.84 (0.77-0.91)	91.3%	0.82 (0.71-0.94)	93.9%	0.85 (0.76-0.94)
**Weight status**						
Underweight/Normal weight<br>Overweight/obesity	90.5%	0.77 (0.70-0.84)	91.3%	0.78 (0.66-0.89)	89.9%	0.77 (0.68-0.86)
**Purchased at least one food**						
Yes<br>No	100.0%	1.00 (0.93-1.00)	100.0%	1.00 (0.89-1.00)	100.0%	1.00 (0.91-1.00)
**Purchased at least one beverage**						
Yes<br>No	100.0%	1.00 (0.93-1.00)	100.0%	1.00 (0.89-1.00)	100.0%	1.00 (0.91-1.00)
**Number of items purchased**						
1 (median)<br>>1	99.1%	0.97 (0.91-1.00)	99.7%	0.98 (0.87-1.00)	98.7%	0.97 (0.89-1.00)
**ITEMS**	***n* = 929**		***n* = 324**		***n* = 605**	
**Item purchased**	98.2%	0.98 (0.97-1.00)	96.3%	0.96 (0.93-0.99)	99.2%	0.99 (0.97-1.00)
**Quantity purchased**	92.0%	0.89 (0.86-0.92)	94.1%	0.91 (0.85-0.97)	90.8%	0.88 (0.85-0.91)

Supplementary Table S2. Decision rules for the elimination of conflicts of observation.

Type of disagreement	Criteria used	Example
**Disagreement on the food item purchased**		
The two observers registered two different food items (*n* = 4).	Conflicting food items were checked for its availability on the corresponding vending site.*	Observer A registered "coffee" and observer B registered "tea". The corresponding vending site did not have tea available. It was assumed that the customer purchased coffee.
The two observers registered the same food item, but with different degrees of specificity (*n* = 13).	The broadest observation was assumed.	Observer A registered "kebab" and observer B registered "chicken kebab". It was assumed that the customer purchased kebab.
The two observers registered a different number of food items (*n* = 7).	The most complete observation was assumed, unless the conflicting item was not available in the corresponding vending site.	Observer A registered "kebab" and observer B registered "kebab" and "coffee". It was assumed that the customer purchased kebab and coffee. If the corresponding vending site did not have coffee available, it would be assumed that the customer purchased kebab.
**Disagreement on the quantity purchased**		
The two observers registered the same food item, but different quantities (*n* = 74).	The average quantity between the two observations was assumed.	Observer A registered 200mL of coffee and observer B registered 150mL of coffee. It was assumed that the customer purchased 175mL of coffee.

*Observations should be excluded in the cases where the availability criterion does not solve the conflict: (1) if the conflicting food items are both available at the corresponding vending site; or (2) if none of the conflicting food items are available at the corresponding vending site. These particular situations did not occur.

Supplementary Table S3. Ready-to-eat foods and beverages purchased by the customers observed in street food and takeaway food vending sites in Bosnia and Herzegovina, overall and by sex, age and weight status.

	Total		Sex					Age[a]					Weight status[a]				
			Male		Female		*p*	<35 years		≥35 years		*p*	Underweight /normal		Overweight /obesity		*p*
	n = 755		*n* = 373		*n* = 382			*n* = 233		*n* = 468			*n* = 494		*n* = 189		
	n	%	*n*	%	*n*	%		*n*	%	*n*	%		*n*	%	*n*	%	
**Foods[b]**	602	79.7	291	78.0	311	81.4	0.246	177	76.0	382	81.6	0.079	391	79.2	155	82.0	0.404
Savoury pastries	189	25.0	106	28.4	83	21.7	**0.034**	44	18.9	131	28.0	**0.009**	120	24.3	51	27.0	0.467
Main dishes	111	14.7	60	16.1	51	13.4	0.289	38	16.3	68	14.5	0.536	69	14.0	34	18.0	0.189
Breads	142	18.8	59	15.8	83	21.7	**0.038**	29	12.4	105	22.4	**0.002**	88	17.8	38	20.1	0.490
Ice-cream, chocolate and confectionery	96	12.7	42	11.3	54	14.1	0.236	41	17.6	46	9.8	**0.003**	68	13.8	23	12.2	0.583
Buns, cakes and cookies	50	6.6	15	4.0	35	9.2	**0.005**	15	6.4	27	5.8	0.725	31	6.3	12	6.4	0.972
Sandwiches	34	4.5	19	5.1	15	3.9	0.439	16	6.9	16	3.4	**0.039**	24	4.9	5	2.7	0.199
Savoury snacks	17	2.3	6	1.6	11	2.9	0.239	6	2.6	10	2.1	0.714	13	2.6	4	2.1	0.699
**Beverages[b]**	241	31.9	135	36.2	106	27.8	**0.013**	79	33.9	144	30.8	0.401	160	32.4	58	30.7	0.670
Water	97	12.9	40	10.7	57	14.9	0.085	34	14.6	56	12.0	0.327	63	12.8	26	13.8	0.727
Soft drinks and industrial juices	91	12.1	54	14.5	37	9.7	**0.043**	41	17.6	41	8.8	**0.001**	65	13.2	18	9.5	0.193
Yoghurt	34	4.5	23	6.2	11	2.9	**0.029**	6	2.6	26	5.6	0.075	19	3.8	11	5.8	0.260
Alcoholic beverages	21	2.8	20	5.4	1	0.3	**<0.001**	0	0.0	21	4.5	**0.001**	13	2.6	5	2.6	0.992
Coffee	2	0.3	1	0.3	1	0.3	0.987	0	0.0	2	0.4	0.318	1	0.2	1	0.5	0.480

[a]For the variables age and weight status, the data presented corresponds to the customers in which there was agreement between observers: *n* = 701 and *n* = 683, respectively; [b]The sum of the values for each sub-category may be higher than the total of the respective category, because the same customer can buy more than one food and/or beverage.
Values in bold represent statistically significant differences according to Pearson's Chi-squared test with a significance level of 0.05.
